# A Method for Variant Agnostic Detection of SARS-CoV-2, Rapid Monitoring of Circulating Variants, and Early Detection of Emergent Variants Such as Omicron

**DOI:** 10.1128/jcm.00342-22

**Published:** 2022-06-29

**Authors:** Eric Lai, Emily B. Kennedy, Jean Lozach, Kathleen Hayashibara, Jeremy Davis-Turak, David Becker, Pius Brzoska, Tyler Cassens, Evan Diamond, Manoj Gandhi, Alexander L. Greninger, Pooneh Hajian, Nicole A. Leonetti, Jason M. Nguyen, K. M. Clair O’Donovan, Troy Peck, Jimmy M. Ramirez, Pavitra Roychoudhury, Efren Sandoval, Cassandra Wesselman, Timothy Wesselman, Simon White, Stephen Williams, David Wong, Yufei Yu, Richard S. Creager

**Affiliations:** a Personalized Science, Burlington, Vermont, USA; b OOMVELT, Lakewood, Ohio, USA; c ROSALIND, San Diego, California, USA; d Thermo Fisher Scientific, South San Francisco, California, USA; e Helix OpCo, San Diego, California, USA; f University of Washington Medical Center, Seattle, Washington, USA; g Fred Hutchinson Cancer Research Center, Seattle, Washington, USA; h Biocomx, Dana Point, California, USA; i Seegene Technologies, Walnut Creek, California, USA; Cepheid

**Keywords:** COVID-19, Delta, genotyping, *in vitro* diagnostics, mutations, Omicron, RT-PCR, SARS-CoV-2, variants, next-generation sequencing

## Abstract

The rapid emergence of SARS-CoV-2 variants raised public health questions concerning the capability of diagnostic tests to detect new strains, the efficacy of vaccines, and how to map the geographical distribution of variants to understand transmission patterns and loads on healthcare resources. Next-generation sequencing (NGS) is the primary method for detecting and tracing new variants, but it is expensive, and it can take weeks before sequence data are available in public repositories. This article describes a customizable reverse transcription PCR (RT-PCR)-based genotyping approach which is significantly less expensive, accelerates reporting, and can be implemented in any lab that performs RT-PCR. Specific single-nucleotide polymorphisms (SNPs) and indels were identified which had high positive-percent agreement (PPA) and negative-percent agreement (NPA) compared to NGS for the major genotypes that circulated through September 11, 2021. Using a 48-marker panel, testing on 1,031 retrospective SARS-CoV-2 positive samples yielded a PPA and NPA ranging from 96.3 to 100% and 99.2 to 100%, respectively, for the top 10 most prevalent World Health Organization (WHO) lineages during that time. The effect of reducing the quantity of panel markers was explored, and a 16-marker panel was determined to be nearly as effective as the 48-marker panel at lineage assignment. Responding to the emergence of Omicron, a genotyping panel was developed which distinguishes Delta and Omicron using four highly specific SNPs. The results demonstrate the utility of the condensed panel to rapidly track the growing prevalence of Omicron across the US in December 2021 and January 2022.

## INTRODUCTION

Since the beginning of the COVID-19 pandemic, variants have emerged that have the potential to evade vaccines, cause diagnostic test performance issues, or cause more severe disease (1–5). Monitoring and surveillance of the genetic lineages of SARS-CoV-2-positive samples are critical to the timely identification of emerging variants (6–8). In November 2021, Omicron, a highly divergent variant harboring multiple mutations, was reported in South Africa (9). While data suggest that Omicron may be associated with reduced risk of hospitalizations compared to Delta, the increased transmissibility and rapid global spread of Omicron highlighted the need for a fast and cost-effective approach for early detection of such variants (10–12).

SARS-CoV-2 genetic lineages in the US are primarily monitored by next-generation sequencing (NGS) on a random selection of approximately 5% percent of SARS-CoV-2-positive samples (13, 14). While extremely accurate at detecting existing circulating variants, NGS does not allow for the timely identification of emerging variants. More focused approaches, such as genotyping using single-nucleotide polymorphism (SNP) assays, offer significant advantages in terms of cost, throughput, and efficient result reporting. Several reports have shown that SNP assays provide rapid turnaround times compared to NGS (15–17). Harper et al. (18) previously demonstrated the utility of a SNP genotyping panel to inexpensively identify SARS-CoV-2 genotypes circulating between March and May 2020. However, previous methods required a two-step approach to genotype a sample: (i) identify a positive sample using reverse transcription PCR (RT-PCR) or an antigen assay; and (ii) genotype the positive sample using a SNP panel. The documented approach seeks to expand upon earlier efforts by developing an assay which simultaneously confirms a SARS-CoV-2-positive sample and identifies its genotype. SARS-CoV-2-positive samples with undetermined genotyping variant classification could provide a prescreened sample set for NGS and potentially allow for early identification of emerging variants.

The National Institutes of Health (NIH) Rapid Acceleration of Diagnostics (RADx) initiative created a Variant Task Force (VTF) in January 2021 to assess the impact of emerging SARS-CoV-2 variants on *in vitro* diagnostic testing (19). In July 2021, the NIH RADx VTF also initiated an effort to develop a SARS-CoV-2 RT-PCR assay for variant agnostic detection of SARS-CoV-2, as well as early detection and monitoring of SARS-CoV-2 variants. Its aims were as follows: (i) identify SARS-CoV-2 markers useful for the detection of SARS-CoV-2-positive samples across all variants; (ii) develop a panel of SNP markers that can be used to accurately assign lineages to SARS-CoV-2-positive samples; and (iii) implement a genotyping approach and platform for the early detection of new and re-emerging variants which signals when markers need to be updated.

This paper outlines the results of the performance validation of the initial genotyping assay and associated marker sets. It also describes how this approach was rapidly adapted to develop a targeted panel of four mutations—three for Omicron and one for Delta—for the purpose of identifying Omicron, and how this Omicron genotyping panel was implemented in several diagnostic labs. The data from this study, as well as associated statistics and trends, are available on a publicly accessible dashboard (20).

## MATERIALS AND METHODS

### Marker selection.

Data analysis for identifying SARS-CoV-2 markers was performed using the Variant Analysis for Diagnostic Monitoring system (ROSALIND, San Diego, CA). Genome sequences and metadata used for the selection of markers in this study were obtained through a Direct Connectivity Agreement for complete daily worldwide downloads from the GISAID EpiCov database (21–23). Sequences not tagged with “is complete” and sequences with an “n_content” of more than 0.05 were excluded. Pairwise whole-genome alignments of all sequences were performed using LASTZ v1.04.02 with the National Center for Biotechnology Information (NCBI) Reference Sequence: NC_045512.2 as the SARS-CoV-2 reference genome (24, 25). The Bioconductor package for genetic variants, VariantAnnotation v1.20.2, was then used for the translation into amino acids in R v3.3.2, and the identification of amino acid substitutions or frameshifts was used to call a unique mutation incident (26, 27).

Selection of the lineages considered for the marker panel was performed by combining the top 100 most frequently reported lineages worldwide for the 120-day period between May 12 and September 11, 2021 (data not shown). A total of 1,200,791 sequences representing 393 lineages were analyzed. The top 10 most unique mutations for each World Health Organization (WHO) label were then identified, and multiple combinations of these unique mutations were evaluated to classify a viral sequence into a WHO label with at least 90% overall accuracy. Additional mutations were added to ensure coverage for the Centers for Disease Control and Prevention (CDC) Variants Being Monitored (VBM), Variants of Interest (VOI), Variants of Concern (VOC), and Variants of High Consequence (VOHC).

The positive-percent agreement (PPA) and negative-percent agreement (NPA) for each marker set compared to NGS was calculated according to the Clinical and Laboratory Standards Institute (CLSI) EP12-A2: User Protocol for Evaluation of Qualitative Test Performance (28). A classifier algorithm was developed to measure the presence, absence, and combinations of mutations to accurately assign the WHO label classification. A dedicated system was established to host the classifier algorithm and provide a web application with application programming interface capabilities for standardized data submission and processing (29). This system was established on a secure virtual private cloud instance on the Google Cloud Platform with the ability to process thousands of specimens per minute.

### Samples.

The 1,128 retrospective clinical samples (1,031 SARS-CoV-2-positive with sequences in GISAID and 97 SARS-CoV-2-negative) used in this investigation were collected from April through December 2021 by Helix OpCo and the University of Washington (UW). The 26,788 prospective clinical samples (all previously determined SARS-CoV-2-positive and including 11,849 with sequence information used to validate the 4-marker panel) were collected from November 2021 through January 2022 by Aegis Sciences Corporation, Helix OpCo, and UW. All clinical samples, retrospective and prospective, were collected predominantly in the states of Washington, Florida, and California. Aegis Sciences Corporation, Helix OpCo, and UW are Clinical Laboratory Improvement Amendments (CLIA)-certified labs participating in the CDC National SARS-CoV-2 Strain Surveillance sequencing program to monitor variant distribution in the US (30). The Pearl independent institutional review board (IRB) gave ethical approval for the use of Aegis Sciences Corporation de-identified remnants of clinical testing. Western Institutional Review Board–Copernicus Group, the institutional review board of record for the Helix Respiratory Registry, gave ethical approval for the use of Helix OpCo de-identified remnants of clinical testing. Use of the UW de-identified excess clinical specimens was approved with a consent waiver by the UW IRB.

### Genotyping assay.

A panel of single well SNP assays, each containing two primers and a duplex of VIC and FAM dye-labeled probes detecting the reference and mutant alleles, respectively, was developed using previously described methods (31). Primers were selected based on mapping to genome regions with a mutation frequency of less than 1%, ensuring that no major polymorphisms interfered with the primers. Primer sets were designed such that amplicon sizes were below 150 bp. Minor groove binder probes were designed to achieve optimal discrimination between the two alleles by taking the position, nucleotide composition, melting temperature (*T_m_*), and the type of allele into consideration. The *T_m_* of the primers ranged from 59 to 62°C and the *T_m_* of the probes ranged from 59 to 65°C. Viral RNA was automatically extracted using the MagMAX Viral/Pathogen II Nucleic Acid isolation kit (Thermo Fisher Scientific, South San Francisco, CA). Within 24 h of extraction, reverse transcription quantitative PCR (RT-qPCR) using the selected panel was performed using the TaqPath 1-Step RT-qPCR Master Mix, CG (Thermo Fisher Scientific) on a QuantStudio 7 Real-Time PCR System or ProFlex 2 × 384-well PCR System (Thermo Fisher Scientific) followed by endpoint data collection using the QuantStudio 7 Real-Time PCR System. Data were analyzed using the TaqMan Genotyper v1.6 software (Thermo Fisher Scientific). The software algorithm used the normalized reported emission (Rn) of VIC (*x* axis) versus the Rn of FAM (*y* axis) from amplification of the reference and mutant alleles to obtain genotype calls in samples with as few as 10 RNA copies per reaction. All the materials required to run this genotyping assay—instruments, consumables, reagents, software, and specific assays for each of the markers—are available commercially (32, 33).

### Next-generation sequencing.

The SARS-CoV-2 sequencing and consensus sequence generation methods used in this study have been previously described (34, 35).

## RESULTS

### Complete marker panel.

The complete genotyping assay panel consisted of 45 lineage specific markers and 3 positivity markers (Table 1).

**TABLE 1:** Set configurations for 48-, 24-, 16-, 12-, and 8-marker sets^*a*^

Nucleotide mutations	AA mutation	Marker set					Classification outcome
		48	24	16	12	8	
*nsp10* gene	None	+	+	+	+	+	SARS-CoV-2 detected
A23403G	S:D614G	+	+	+	+		
N gene SC2	None	+					
T16176C	None	+	+	+	+	+	Alpha
A21801C	S:D80A	+	+	+	+	+	Beta
A22812C	S:K417T	+	+	+	+	+	Gamma
C21618G	S:T19R	+	+	+	+	+	Delta
C22995A	S:T478K	+	+	+	+	+	Delta
T7424G	orf1ab:F2387V	+	+	+	+	+	Lambda
A13057T	None	+	+	+	+	+	Mu
G22018T	S:W152C	+	+	+	+		Epsilon
A16500C	orf1b:Q1011H	+	+	+	+		Iota
T22917A	S:L452Q	+	+	+	+		Lambda
A11456G	orf1ab:I3731V	+	+	+			Delta
A28699G	None	+	+	+			Eta
G23593C	S:Q677H	+	+	+			Eta
A24775T	S:Q1071H	+	+	+			Kappa
TACATG21765—	S:HV69–	+	+				Alpha
TTA21991—	S:Y144-	+	+				Alpha (when combined with T16176C)
CTTTACTTG22281—	S:LLA241—	+	+				Beta (when combined with A21801C)
T733C	None	+	+				Gamma
T22917G	S:L452R	+	+				Delta (or Epsilon when combined with G22018T)
A22320G	S:D253G	+	+				Iota (when combined with A16500C)
G23012C	S:E484Q	+	+				Kappa (when combined with A24775T)
C27925A	ORF8:T11K	+	+				Mu
G22132T	S:R190S	+					Gamma
C23604G	S:P681R	+					Delta
C25469T	ORF3a:S26L	+					Delta
G5629T	None	+					CDC VBM, VOI, VOC, or VOHC
C21614T	S:L18F	+					
T21615G	S:L18R	+					
C21638T	S:P26S	+					
G21770T	S:V70F	+					
A21801G	S:D80G	+					
G22335T	S:W258L	+					
G22813T	S:K417N	+					
T22882G	S:N440K	+					
C22995G	S:T478R	+					
G23012A	S:E484K	+					
A23063T	S:N501Y	+					
A23064C	S:N501T	+					
A23592C	S:Q677P	+					
G23593T	S:Q677H	+					
C23604A	S:P681H	+					
C23664T	S:A701V	+					
G24410A	S:D950N	+					
T27206C	ORF6:F2S	+					
T28226C	None	+					

a CDC, Centers for Disease Control and Prevention; VBM, Variant Being Monitored; VOI, Variant of Interest; VOC, Variant of Concern; VOHC, Variant of High Consequence.

### Variant agnostic positivity markers.

The 3 variant agnostic markers selected to detect SARS-CoV-2 positivity were as follows: (i) the S gene: D614G mutation, a nonsynonymous mutation present in nearly all SARS-CoV-2 sequences which results in the replacement of aspartic acid with glycine at position 614 of the viral spike protein; (ii) a conserved sequence in *nsp10* (nucleotides 13,025 to 13,441); and (iii) a conserved sequence identified by the CDC in the N gene SC2 region (nucleotides 29,461 to 29,482) (36).

A total of 1,128 retrospective samples (1,031 SARS-CoV-2-positive with sequences in GISAID and 97 SARS-CoV-2-negative) collected between April and December 2021 were evaluated using the variant agnostic positivity markers (Table 2). The combined markers were detected in all but 7 of the 1,031 SARS-CoV-2-positive samples. The quantification cycle (*Cq*) range for these 7 positive samples was 22 to 33. The PPA using any combination of 2 or more markers was greater than or equal to 98.9% with the criteria being that 1 marker detected was enough to make a positive call. Additionally, the PPA using 1 marker was greater than or equal to 96%. There were no false-positive results (data not shown).

**TABLE 2:** Variant agnostic positivity markers *in vitro* performance

No. of markers	Variant agnostic marker			Positive calls (*n*)^*a*^	PPA (%)
	S:D614G	*nsp10* gene	N gene SC2		
3	+	+	+	1,024	99.3
2	+	+		1,020	98.9
	+		+	1,021	99
		+	+	1,023	99.2
1	+			993	96.3
		+		1,018	98.7
			+	990	96
0				7	0.7

a Total SARS-CoV-2-positive samples: 1,031.

### Lineage assignment.

The performance of the genotyping assay panel and the associated classifier was determined by *in silico* and *in vitro* studies with retrospectively collected SARS-CoV-2 specimens.

A bioinformatics simulation was performed using GISAID SARS-CoV-2 sequence data from the first week of each month from November 2020 to October 2021. A total of 323,148 GISAID sequences were analyzed. With the 48-marker set, for the top 10 most prevalent WHO lineages as of September 11, 2021 (Alpha, Beta, Gamma, Delta, Epsilon, Eta, Iota, Kappa, Lambda, and Mu), simulated PPA ranged from 80.7% to 99.9% and simulated NPA ranged from 98.1% to 100% (Table 3). The performance for the Kappa variant was impacted by reporting from Asia and Oceania, where many Kappa-positive samples were misclassified as Delta.

**TABLE 3:** Performance of 48-marker set *in silico* classifier^*a*^

Lineage	48 Markers	
	Simulated PPA (%)	Simulated NPA (%)
Alpha	99.9	98.8
Beta	97.7	100
Gamma	99	100
Delta	99.8	98.1
Epsilon	96.1	100
Eta	96.2	100
Iota	98.4	100
Kappa	80.7	100
Lambda	98.1	100
Mu	98.8	100

a PPA, positive percent agreement; NPA, negative percent agreement.

We next determined the clinical performance of the genotyping assay and classifier. The 1,031 SARS-CoV-2-positive samples were genotyped and classified with the 48 markers shown in Table 1. The classifications were then compared to the Phylogenetic Assignment of Named Global Outbreak Lineages (Pango) assignment based on the whole-genome sequences in the GISAID database (Table 4) (37). For the top 10 WHO lineages, the PPA ranged from 96.3% to 100% and the NPA ranged from 99.2% to 100%. The classifier categorized an additional 78 samples as undetermined (data not shown). Pango assigned 77 of these samples to 14 lineages for which the genotyping assay does not include specific markers (Zeta, B.1, B.1.1.507, B.1.2, B.1.221, B.1.241, B.1.517, B.1.596, B.1.609, B.1.625, B.1.628, B.1.634, B.1.637, and C.36.3), and did not classify 1 of these samples.

**TABLE 4:** Performance of 48-marker set *in vitro* classifier

Pango assignment	Result	Classifier call (*n*)		Percent agreement (%)^*a*^
		Positive	Negative	
Alpha	Positive	122	1	99.2
	Negative	7	908	99.2
Beta	Positive	13	0	100
	Negative	0	1,018	100
Gamma	Positive	109	0	100
	Negative	0	922	100
Delta	Positive	456	5	98.9
	Negative	1	570	99.8
Epsilon	Positive	78	3	96.3
	Negative	3	950	99.7
Eta	Positive	27	0	100
	Negative	0	1,004	100
Iota	Positive	82	0	100
	Negative	1	949	99.9
Kappa	Positive	1	0	100
	Negative	0	1,030	100
Lambda	Positive	2	0	100
	Negative	0	1,029	100
Mu	Positive	54	0	100
	Negative	0	977	100

a Positive or negative percent agreement.

### Marker reduction.

To optimize assay performance in terms of sample input, reductions of the 48-marker panel were explored. We assessed the performance of 24-, 16-, 12-, and 8-marker sets which were defined based on mutation combination performance and targeted lineage prevalence during the 120-day period between May 12 and September 11, 2021 (Table 5). Each of the panels also included 2 of the variant agnostic positivity markers (*nsp10* gene and S:D614G), which were used as assay internal controls. The 48-, 24-, and 16-marker sets identified the top 10 WHO lineages, while the 12- and 8-marker sets identified 8 and 6 of the top 10 WHO lineages, respectively.

**TABLE 5:** Performance of 48-, 24-, 16-, 12-, and 8-marker set *in vitro* classifiers^*a*^

Classifier call	48 markers		24 markers		16 markers		12 markers		8 markers	
	PPA (%)	NPA (%)	PPA (%)	NPA (%)	PPA (%)	NPA (%)	PPA (%)	NPA (%)	PPA (%)	NPA (%)
Alpha	99.2	99.2	99.2	99.2	98.4	99.1	98.4	99.1	98.4	99.1
Beta	100	100	100	100	100	100	100	100	100	100
Gamma	100	100	100	100	100	100	100	100	100	100
Delta	98.9	99.8	98.7	99.6	98.7	99.6	98.7	99.6	98.9	99.8
Epsilon	96.3	99.7	96.3	99.7	96.3	99.7	96.3	99.7	–^*b*^	91.5
Eta	100	100	100	100	100	100	–^*b*^	97.3	–^*b*^	97.3
Iota	100	99.9	100	99.9	100	99.9	100	99.9	–^*b*^	91.3
Kappa	100	100	100	100	100	100	–^*b*^	99.9	–^*b*^	99.9
Lambda	100	100	100	100	100	100	100	100	100	100
Mu	100	100	100	100	100	100	100	100	100	100

a PPA, positive percent agreement; NPA, negative percent agreement.

b Cannot call.

**FIG 1:**
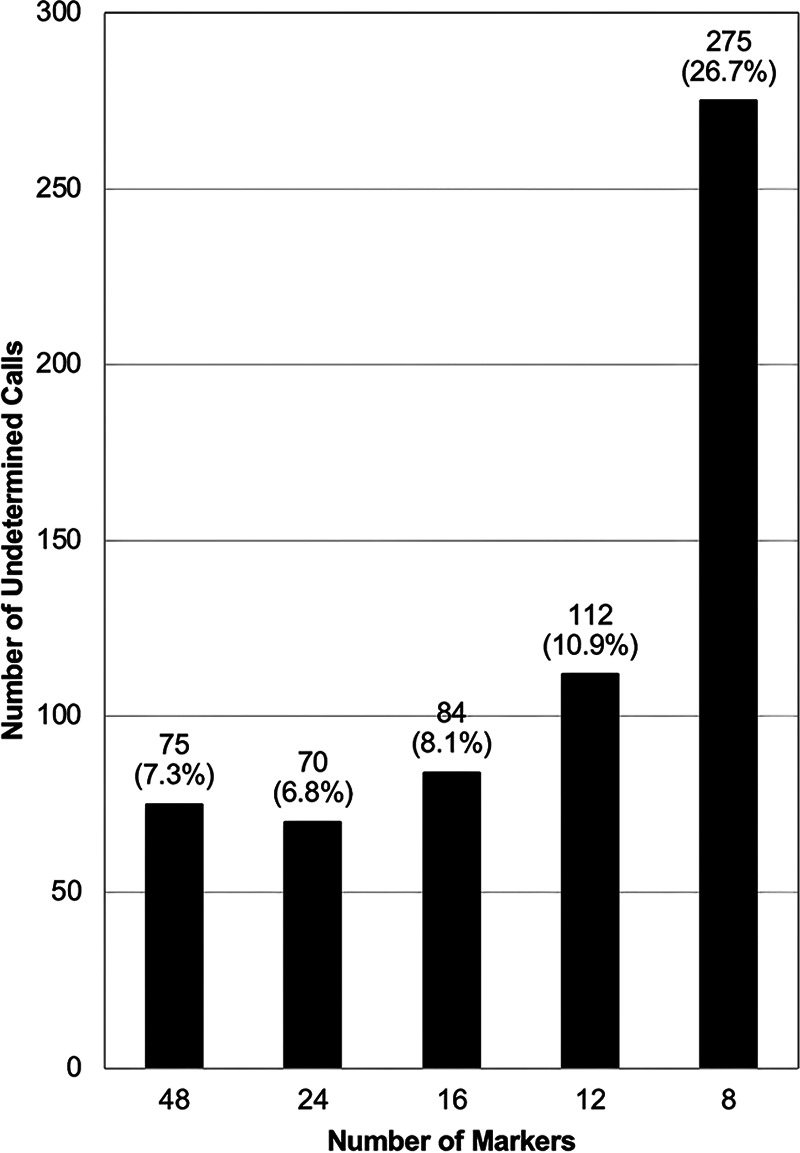
Number of undetermined calls in *in vitro* classifier performance analysis.

The identification of samples that could not be determined by the classifier was further investigated. The number of undetermined samples for each marker set are shown in Fig. 1. The percentage of undetermined samples ranged from approximately 7% to 11% for the 12- to 48-marker sets and increased dramatically to 27% with the 8-marker panel. The shift from a 48- to 8-marker set is associated with 6-fold increased throughput and 6-fold cost reduction, assuming a fixed cost per single-well SNP assay.

### Early detection of new variants.

**FIG 2:**
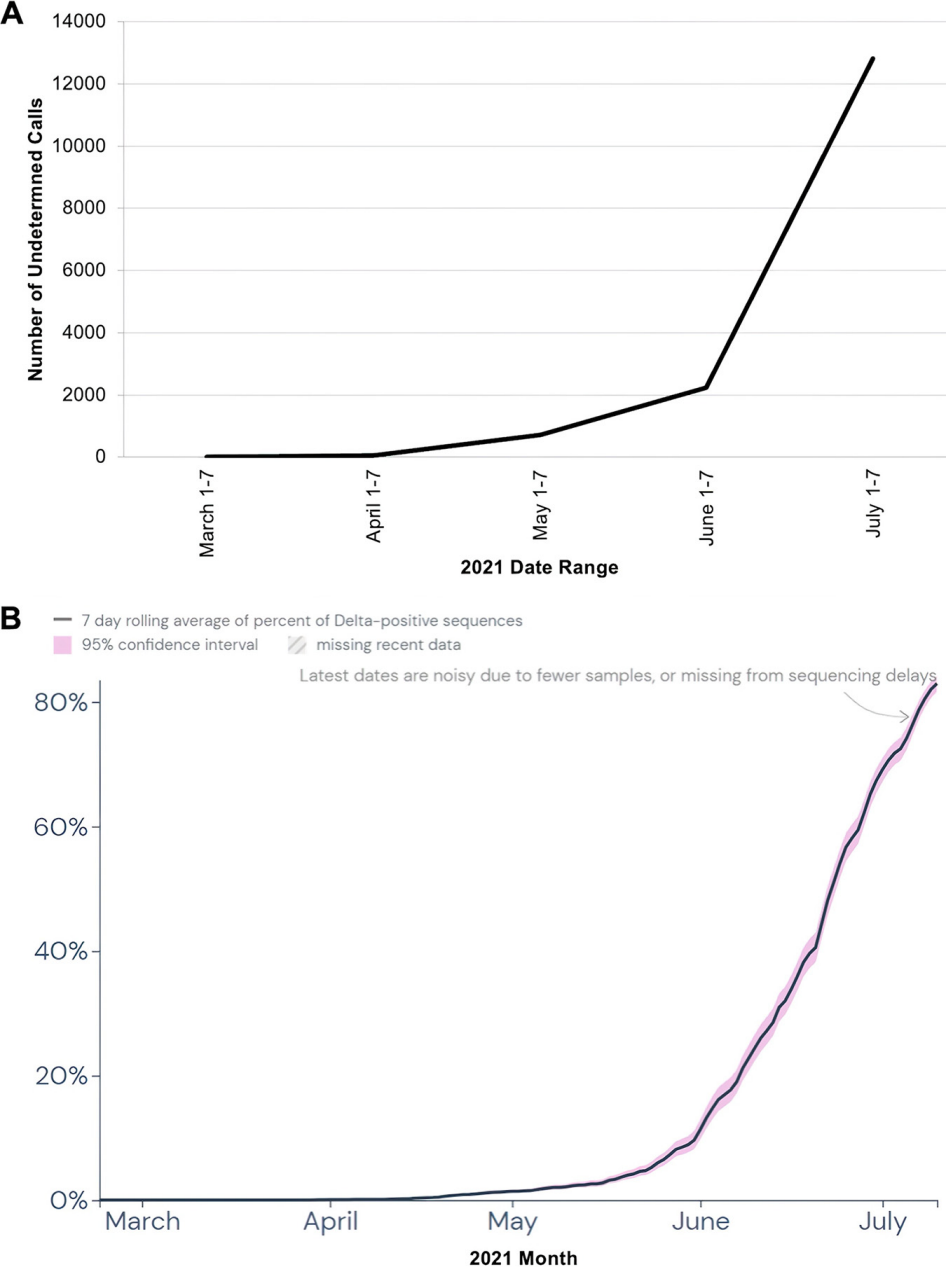
Undetermined calls using 12-marker set without Delta-specific markers (A) compared to average daily Delta prevalence in the US from March through July 2021 (B). Panel B image courtesy of outbreak.info (40).

One of the aims of this study was to develop a genotyping approach for the early detection of emerging variants. An increase in the number of undetermined calls by the classifier provides a signal for focused sequencing of those samples, potentially allowing early detection of new variants. To test this hypothesis, a bioinformatics simulation was performed using a modification of the 12-marker panel. The 2 Delta-specific markers were removed to simulate what would have been observed before and during the emergence of the Delta variant. The number of undetermined calls in the first week of each month from March through July 2021 is shown in Fig. 2. Panel A. The 12-marker set without the 2 Delta-specific markers was able to assign lineages to all positive samples in GISAID for North America in November and December 2020 (data not shown). The numbers of undetermined calls were 5 of 16,901, 7 of 17,542, and 1 of 21,869 in January 2021, February 2021, and March 2021, respectively (all less than 0.05%). In April 2021, the number increased to 51 of 40,398 (0.1%), followed by a rapid increase over the following 3 months to 12,825 of 16,615 (77.2%) undetermined calls in July 2021. Then, results were compared to the average daily Delta prevalence in the USfrom March to July 2021 as reported by the CDC (Fig. 2B). The prevalence data for the emerging Delta variant mirrors the rate of increase in undetermined calls over the same period.

### Addition of Omicron markers.

In response to the emergence of the Omicron variant in November 2021 in South Africa, an assay was rapidly developed containing markers specific for this variant. Sequence analysis of the first 132 Omicron sequences revealed 3 markers—ORF1ab:A2710T, ORF1ab:T13195C, and S:T547K—which were found in high percentages of these sequences. Based on *in silico* modeling, there was greater than 99% concurrence between the Pango assignment based on the GISAID sequence and the combined 3 markers (data not shown). Subsequently, a genotyping assay consisting of the 3 Omicron-specific markers and 1 Delta-specific marker (S:T19R) was developed. Variant agnostic positivity markers were excluded from the 4-marker set evaluated in this study using previously determined SARS-CoV-2-positive samples but could be readily added for simultaneous interrogation of positivity and genotyping.

A total of 11,849 previously determined SARS-CoV-2-positive samples were collected and genotyped between November 2021 and January 2022 (Table 6). Sequencing confirmed that these samples consisted of 8,870 Omicron, 2,630 Delta, and 105 samples from 12 other lineages (A, B, B.1, B.1.1, B.1.1.161, B.1.1.26, B.1.1.305, B.1.1.519, B.1.350, B.1.551, B.1.609, and B.3.1), as well as 244 samples that were not classified by Pango and thus excluded from analysis. The 4-marker panel for Omicron genotyping correctly identified 99.8% of the Omicron samples and 93.8% of the Delta samples. The 4-marker panel incorrectly identified 7 Delta samples as Omicron and classified 156 Delta samples as undetermined. The panel also classified 14 Omicron samples as undetermined. The 105 samples representing other lineages were classified as 5 Delta, 92 Omicron, and 8 undetermined.

**TABLE 6:** Performance of 4-marker set *in vitro* classifier

Classifier call	Result	Pango assignment		Percent agreement^*a*^
		Positive	Negative	
Omicron	Positive	8,856	99	99.8
	Negative	14	2,636	96.4
Delta	Positive	2,467	5	93.8
	Negative	163	8,970	99.9

a Positive or negative percent agreement.

### Prevalence of Omicron in the US in December 2021 and January 2022.

**FIG 3:**
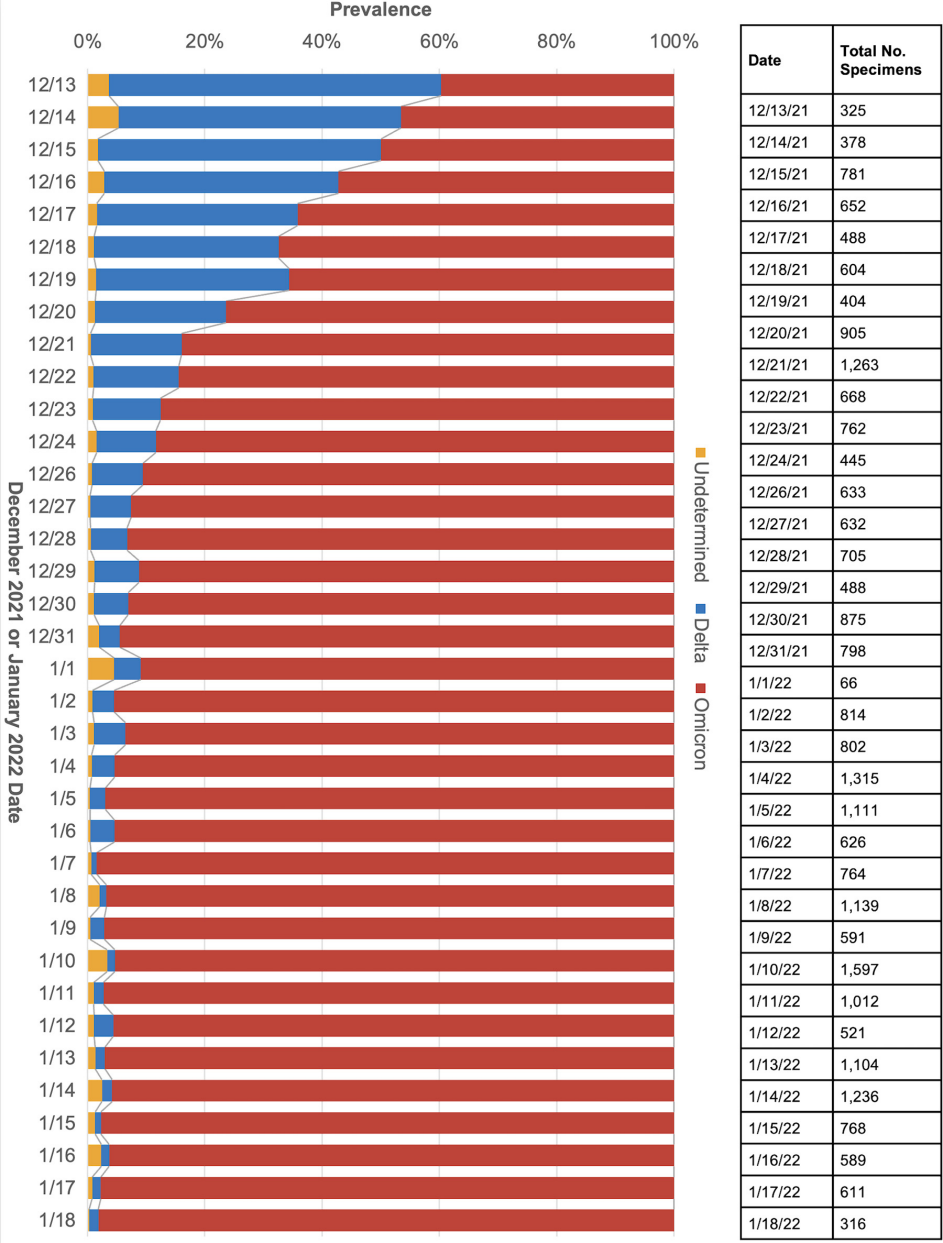
Four-marker set *in vitro* Delta and Omicron classifier calls.

We next deployed the 4-marker panel in three CLIA-certified labs and genotyped an additional 14,939 previously determined SARS-CoV-2-positive samples, for a total of 26,788 SARS-CoV-2-positive samples collected between November 2021 and January 2022. Using the 4-marker panel, it was determined that the relative prevalence of the Omicron variant grew from approximately 40% on December 13, 2021, to more than 90% 2 weeks later on December 27, 2021, while Delta decreased from approximately 60% to less than 10% over the same period. By January 18, 2022, the prevalence of the Omicron variant further increased to over 95% and Delta further decreased to less than 5% (Fig. 3).

## DISCUSSION

The SARS-CoV-2 virus continues to mutate at an unprecedented scale. NGS is the main method used to track the emergence of new variants; however, NGS technology is expensive and current reporting to GISAID and national regulatory authorities typically takes several weeks (15–17). As of January 27, 2022, there were 7,457,886 sequences in GISAID. Nearly 80% of these sequences were deposited by 10 countries (US, United Kingdom, Germany, Denmark, Canada, France, Japan, Sweden, Switzerland, and India), and 90% by two continents (Europe and North America) (21–23). With approximately 400 million COVID-19 cases reported since the start of the pandemic, this translates to geographically limited sequencing of less than 2% percent of available samples (38). Clearly, there is a need for a more widely accessible and efficient method to detect the emergence of new variants.

The use of a high-throughput, low-cost RT-PCR genotyping panel was shown by previous authors to enable rapid identification of circulating SARS-CoV-2 variants. Neopane et al. (39) demonstrated good concordance between their assay and sequencing for variants circulating between March and July 2021. Out of 150 SARS-CoV-2-positive specimens, 69 (46%) were B.1.617.2, 49 (32.7%) were B.1.1.7, 4 each were P.1 and P.2 (2.7% each), 3 were B.1.526 (2%), and 2 each were B.1.351 and B.1.427 (1.3% each). An additional 17 (11.3%) had a mutation only in D614G. However, 13 of the 14 SNPs used in the panel by Neopane et al. were from the S gene, and many of these mutations are now found in different SARS-CoV-2 lineages (40).

Korukluoglu et al. (41) described a one-step RT-qPCR assay to detect N501Y and HV69-70del using allele-specific forward primers, reserving ORF1ab as an internal control. This was a relatively small study with 165 specimens, and the authors observed 100% concordance with results of Sanger sequencing and NGS. Vogels et al. (42) reported on a RT-qPCR assay to detect ORF1a SGF3675-3677del and spike HV69-70del. This assay was concordant with 76 sequenced specimens. Perchetti et al. (43) utilized a two-step approach combining the CDC-based laboratory-developed RT-qPCR and the Thermo Fisher TaqPath COVID-19 RT-PCR to identify B.1.1.7 variants. However, the Perchetti et al. approach employed labor-intensive droplet digital PCR (ddPCR) and depended on the S gene dropout, which is now known to occur in multiple variants (44–48).

Harper et al. (18) developed a genotyping panel to detect variants identified from SARS-CoV-2 sequences surveyed between March and May 2020 and tested this on 50 stored qRT-PCR positive SARS-CoV-2 clinical samples. They initially identified 22 SNPs that could discriminate 15 different genotypes, but subsequent analysis on a larger sequence data set indicated that their approach required 51 markers to maximize sample discrimination. The largest nucleic acid amplification test-based genotyping series thus far was a national effort in France recently reported by Haim-Boukobza et al. (49) The authors used two separate assays to screen for the HVdel69 to 70 and N501Y mutations in 35,208 samples. However, this approach was unable to genotype 19% of the samples.

We sought to expand upon these previous studies by analyzing more than 7.5 million sequences from GISAID to select target SNPs. Our study goals were 3-fold: (i) identify SARS-CoV-2 markers useful for the detection of SARS-CoV-2-positive samples across all variants; (ii) develop the smallest set of SNP markers that can be used to accurately assign lineages to SARS-CoV-2-positive samples (PPA ≥ 95% compared to NGS); and (iii) implement a genotyping approach and platform for the early detection of new and re-emerging variants that signals when markers need to be updated. There may be a bias in our results given that the retrospective and prospective clinical samples used to validate assays originated primarily from Washington, Florida, and California; however, subsequent studies using samples from a broader swath of states showed similar results (data not shown), and national and global knowledge of circulating strains at the time of this study suggest that any bias would be minimal.

This report identifies three variant agnostic markers that can detect SARS-CoV-2-positive samples with high PPA and NPA compared to NGS. These markers are present in almost all the SARS-CoV-2 samples that were sequenced and should be considered in the development of new assays. This study also demonstrates that some marker combinations are highly specific for certain variants. Routine use of these genotyping markers could provide early warning that a new or re-emergent variant is circulating. Importantly, genotyping with this assay is quick and efficient, enabling result reporting in 1 to 2 days, compared to 10 to 14 days with NGS, and for a fraction of the cost. As such, genotyping can be used to monitor a higher percentage of SARS-CoV-2-positive samples than the 5% percent random sampling by sequencing currently practiced in the US. Samples which cannot be assigned to a known variant would be prime candidates for sequencing. Finally, the study demonstrates that the Omicron variant can be identified with high precision with three markers. Incorporating Omicron-specific markers with the markers defined to detect previous variants can provide a framework for the detection of the next new variant.

The genotyping markers for Omicron effectively highlighted the transition from Delta to Omicron as the dominant variant. As illustrated in this report, a static marker set with the Delta-specific markers omitted experienced a significant decline in accuracy within four months of the emergence of Delta in the US. To prevent a loss of marker accuracy, this study demonstrated an approach for detecting new and emerging variants using a classifier algorithm for recurring analysis of active marker sets across regional and global GISAID sequence data. As emerging variants develop, anomalies in classifier calls and the resulting discordance with sequencing classification will continue to highlight the need for marker modifications. With the genotyping assay described herein, addition or subtraction of markers is straightforward. Each SNP marker, including variant agnostic positivity markers, is interrogated in a separate, individual well so that changing one marker has no impact on the performance of other markers in the set. The genotyping assay can be customized to include one or two positivity markers, as well as lineage assignment markers appropriate for the current variant landscape, to simultaneously confirm a SARS-CoV-2-positive sample and identify its genotype. Standardized performance metrics, such as limit of detection (LoD), sensitivity and specificity of custom successor assays, will be easy to establish. A formal LoD was outside the scope of this study, but positivity and genotype calls were obtained for samples with as few as 10 RNA copies per well.

A retrospective review of the emergence of Delta in the US showed that as this variant grew in prevalence, so too did the number of undetermined calls returned by the classifier algorithm. Thus, the classifier algorithm effectively assesses the accuracy of current marker sets based on daily analysis of new viral sequences added to GISAID, creating an adaptive and closed-loop process for low-cost, rapid monitoring of circulating variants and detection of emerging variants. Indeed, the authors recently created a free, live dashboard of a real-time genotyping platform illustrating the symbiotic nature of using genotyping markers in conjunction with targeted sequencing (20). An uptick in SARS-CoV-2-positive samples with undetermined genotyping variant classification will trigger targeted sequencing of this subset of samples to determine whether they represent a novel variant for which genotyping markers should be developed and incorporated. This real-time tracking tool will become increasingly powerful as more public health and private testing labs adopt this genotyping approach and contribute data.
